# Expression of DNA damage response proteins and complete remission after radiotherapy of stage IB–IIA of cervical cancer

**DOI:** 10.1038/sj.bjc.6603153

**Published:** 2006-05-09

**Authors:** C Beskow, L Kanter, Å Holgersson, B Nilsson, B Frankendal, E Åvall-Lundqvist, R Lewensohn

**Affiliations:** 1 Department of Gynaecologic Oncology, Radiumhemmet, Karolinska University Hospital, Solna, SE-171 76 Stockholm, Sweden; 2 Department of Pathology and Cytology, Karolinska University Hospital, Solna, SE-171 76 Stockholm, Sweden; 3 Department of Oncology and Pathology, Karolinska University Hospital, Solna, SE-171 76 Stockholm, Sweden; 4 Unit of Medical Radiation Biology, Department of Oncology and Pathology, Karolinska Institutet, SE-171 76 Stockholm, Sweden

**Keywords:** cervical cancer, radiosensitivity, DNA-PK, p53, p21, Mdm2

## Abstract

The primary aim of this study was to investigate if the expression of the DNA damage identifying protein DNA-PKcs known to be involved in DNA repair after treatment with ionising radiation can be used as a predictive marker for radiotherapy (RT) response in cervical cancer. Formalin-fixed primary tumour biopsies from 109 patients with cervical cancer, FIGO-stage IB–IIA, treated with preoperative brachytherapy followed by radical surgery were analysed by immunohistochemistry. In addition, correlation studies between early pathological tumour response to radiation and expression of Ku86, Ku70, Mdm-2, p53 and p21 in primary tumours were also performed. We found that tumour-transformed tissue shows positive immunostaining of DNA-PKcs, Ku86 and Ku70, while non-neoplastic squamous epithelium and tumour-free cervix glands show negative immunoreactivity. Expression of DNA-PKcs positively correlated with both Ku86 and Ku70, and a statistically significant correlation between the Ku subunits was also found. After RT, 85 patients demonstrated pathologic complete remission (pCR), whereas 24 patients had residual tumour in the surgical specimen (non-pCR). The main finding of our study is that there was no correlation between the outcome of RT and the expression of DNA-PK subunits. Positive p53 tumours were significantly more common among non-pCR cases than in patients with pCR (*P*=0.031). Expression of p21 and Mdm-2 did not correlate with the outcome of RT.

Early stage cervical cancer can be successfully treated by radiotherapy (RT). We have shown that pathologic complete remission (pCR) after preoperative brachytherapy as compared to incomplete pathologic remission (non-pCR) is a strong predictive factor for long-term survival (Beskow *et al*, 2002).

The primary action of ionising radiation is to produce DNA damage, leading to cell death. At the cellular level, DNA double strand breaks (DSBs) are considered as the most lethal lesions and sensitivity to ionising radiation will depend on the DSB repair efficacy possessed by the cell. Nonhomologous end joining (NHEJ) is a major pathway for repair of DNA DSBs in mammalian cells (Hefferin and Tomkinson, 2005). The initial step of NHEJ involves binding of the DNA-dependent protein kinase, DNA-PK, which holds the ends to be joined in physical proximity, permitting modifications of the broken ends to make them compatible before ligation (DeFazio *et al*, 2002). DNA-PK is composed of a 465 kDa catalytic subunit, DNA-PKcs, and a heterodimer of Ku86 and Ku70, which acts as a DNA targeting and regulatory component of the complex (Mimori and Hardin, 1986; Dynan and Yoo, 1998). DNA-PKcs is a serine/threonine protein kinase belonging to the phosphatidylinositol-3 kinase-related kinase family, which include other large proteins involved in DNA repair signalling, such as ATM and ATR (Shiloh, 2003). Genetic mutations in the ATM or DNA-PKcs gene result in immunodeficiency, cancer susceptibility and hypersensitivity to ionising radiation (Lavin and Shiloh, 1997; Rotman and Shiloh, 1999). However, in contrast to ATM, DNA-PKcs deficiency has not been described in humans, as in lower species (Blunt *et al*, 1995; Wiler *et al*, 1995; Shin *et al*, 1997; Meek *et al*, 2001; Ding *et al*, 2002). In human tumour cell lines of glioblastoma origin DNA-PKcs has been shown to be important for their response to radiation (Allalunis-Turner *et al*, 1993). Also in lung cancer cell lines a correlation between content of DNA-PKcs and radiation sensitivity has been demonstrated (Sirzen *et al*, 1999). The primary aim of our study was to evaluate a possible role between the frequency of tumour cells in cervical cancer staining positively for DNA-PKcs and tumour response to RT. As a deficiency in Ku86 or Ku70 also has been shown to confer increased sensitivity to ionising radiation (Taccioli *et al*, 1994; Jeggo *et al*, 1995), parallel staining for these two subunits of DNA-PK was performed as part of secondary aims.

Also proteins downstream of DNA-PK would be of potential interest to evaluate in the same direction. DNA-PK has been shown to phosphorylate several nuclear DNA-binding proteins *in vitro,* including the transactivation domain of p53 (Lees-Miller *et al*, 1992). The tumour suppressor gene p53 plays a critical role in the cellular response to ionising radiation (Kastan *et al*, 1991). One of the results of p53 activation is p21 synthesis and accumulation, eventually causing G_1_ arrest (el-Deiry *et al*, 1993; Harper *et al*, 1993). If the DNA repair enzymes succeed in repairing the DNA DSBs, activation of p53 ceases, p21 is degraded and the cell is then free to proceed through the cell cycle. If the DNA repair machinery fails, activated p53 can drive the cell to apoptosis (Vousden, 2000). Regulation of p53 is partially carried out by Mdm-2, which binds to p53 and thereby renders it both inactive and available for degradation (Unger *et al*, 1999). Phosphorylation of p53 leads to release from Mdm-2 control and makes p53 transcriptionally active (Shieh *et al*, 1997). In addition, disassociation between p53 and Mdm-2 may be achieved by phosphorylation of Mdm-2 and previously, it was demonstrated that DNA-PK can phosphorylate Mdm-2, rendering Mdm-2 unable to inhibit p53 transactivation (Mayo *et al*, 1997). When DNA repair is accomplished, DNA-PK activity is abolished, resulting in newly synthesised Mdm-2 protein that is unphosphorylated and capable of binding p53, allowing cell cycle progression.

In carcinoma of the cervix, there are indications pointing to intrinsic radiosensitivity of the tumour as a determinant of outcome following RT (West *et al*, 1997). The possibility to measure radiosensitivity in the primary tumour would be of great importance when deciding optimal treatment regimen for the individual patient.

In the present study, we analysed primary tumour biopsies from patients with cervical cancer, treated with preoperative brachytherapy followed by surgery. In addition to our primary goal to assess for a relation between DNA-PKcs and response to RT as secondary goals the expression of Ku86, Ku70, Mdm-2, p53 and p21 were analysed for their impact on response to RT. To assess protein expression we used immunohistochemistry (IHC) staining providing us with a frequency of stained tumour cells for each case analysed. These data were correlated with treatment response defined as pathological complete tumour remission after RT.

## MATERIALS AND METHODS

### Sample size determination

Our primary end point was pathological complete tumour remission. Our hypothesis was that low percentage immunostaining (below the median) of DNA-PKcs in primary tumour would result in pCR after RT to a greater extent than high (above the median) immunopositivity of DNA-PKcs. Our null hypothesis was that no difference in pathological complete tumour remission prevailed in that respect. If the patients with low DNA-PKcs showed a rate of complete pathological tumour remission of 45% and the ‘high’ group had 20% complete tumour remission we should need 2 × 43=86 patients to detect that difference. This is true if the significant level is 5%, the power is 80% and with a one sided hypothesis.

### Patients

Pretreatment biopsy specimens were obtained from 109 patients with cervical carcinoma FIGO stage IB-IIA, treated at the Department of Gynaecologic oncology, Radiumhemmet, during January 1989 to December 1991. Age at time of diagnosis varied between 23 and 75 years, with a mean age of 44 years. Median follow-up for patients alive and not alive were 69 months (range 50–109 months) and 19 months (range 0–87 months), respectively. There were 25 patients with small tumours (<2 cm), 47 patients with large tumours (⩾4 cm) and 37 patients had tumours of intermediate size (2–<4 cm). Tumour characteristics, such as stage distribution, tumour size, histopathological type and grade in relation to the degree of tumour remission after RT is shown in Table 1. Preoperative treatment included two uterovaginal insertions with a 3-week interval. Surgery according to the Wertheim–Meig procedure was performed 4 weeks after the second insertion. Brachytherapy was given according to the ‘Stockholm method’, described elsewhere (Beskow *et al*, 2002). The remote after-loading technique with caesium was used in 69 patients giving a mean total dose of 43 Gy and a mean dose rate to point A of 1.35 Gy h^−1^. In all, 40 patients were treated with the manual technique using radium applicators with a calculated total uterovaginal mean dose of 6500 milligram-hours of radium (mghRa) and a retrospectively estimated dose rate to point A of 1.1 Gy h^−1^. Tumour response did not differ significantly between the two techniques. The use of biopsies and surgical specimens for the present study was approved by the Ethics Committee at the Karolinska Institute. Informed consent were received from all patients alive at time of analysis.

### Assessment of early pathological response

Evaluation of RT response was performed by histopathological examination of the formalin-fixed surgical specimen within 1 week after surgery. All specimens were re-evaluated by a senior pathologist. Tumour remission was assessed in the surgical specimen and was classified as pCR if no morphological intact tumour cells were found or as non-pCR if residual tumour was detected.

### Immunohistochemistry

Immunostaining was preformed using the ABC-technique (Vector Laboratories, Elite Standard Kit. Cat. PK-6100, Burlingame, CA, USA). Paraffin sections (4 *μ*m), placed on Super Frost Plus slides, were dewaxed and rehydrated. The slides were boiled in citrate buffer pH 6.0, in a microwave oven for 12 min at 800 W and 20 min at 250 W. The slides were then cooled and rinsed with phosphate buffer saline (PBS). After blocking with 1% BSA, the proteins were detected by using mouse monoclonal antibodies to DNA-PKcs 1 : 100, Ku86 1 : 200, Ku70 1 : 200 (Neo Markers, Union City, CA, USA), p53 1 : 100 (clone DO-7, DAKO, Kyoto, Japan), p21 1 : 50 (clone EA10, Oncogene research products, Cambridge, MA, USA), and Mdm-2 at 1 : 50 (SMP14, Santa Cruz Biotechnology, Santa Cruz, CA, USA). The protein primary antibody was then detected by incubation with a biotinylated secondary antibody and ABC complex (Vector Laboratories), using diaminobenzedine substrate as a chromogene. The specimens were counterstained with haematoxylin. Staining evaluation was performed in a blinded fashion, without prior knowledge of the clinical data or outcome for the individual patient. Counting was carried out on four representative areas in a light microscope. In every specimen 500 tumour cells were counted and the percentage of positive stained cells was determined. Intensity of immunostaining was judged as low, intermediate or strong. Morphologically normal cells in each specimen served as internal negative control. The intraobserver reproducibility was tested and showed significant correlation between the two series of evaluations (*r*=0.95, *P*<0.001).

### Statistical analysis

Statistical tests on data were based on *χ*^2^. When comparison between high and low protein expression was assessed, a high protein level was defined as a percentage of stained cells above the median value and a low protein level was defined as a percentage of stained cells below the median value. Correlation coefficient of Spearman (*r*_s_) was calculated to compare expression of proteins. Survival analyses on continuous background factors were calculated by Cox regression bivariate analysis.

Pearson correlation test was used to test intraobserver variation.

## RESULTS

### DNA damage response proteins in cervical carcinoma

**Figure 1:**
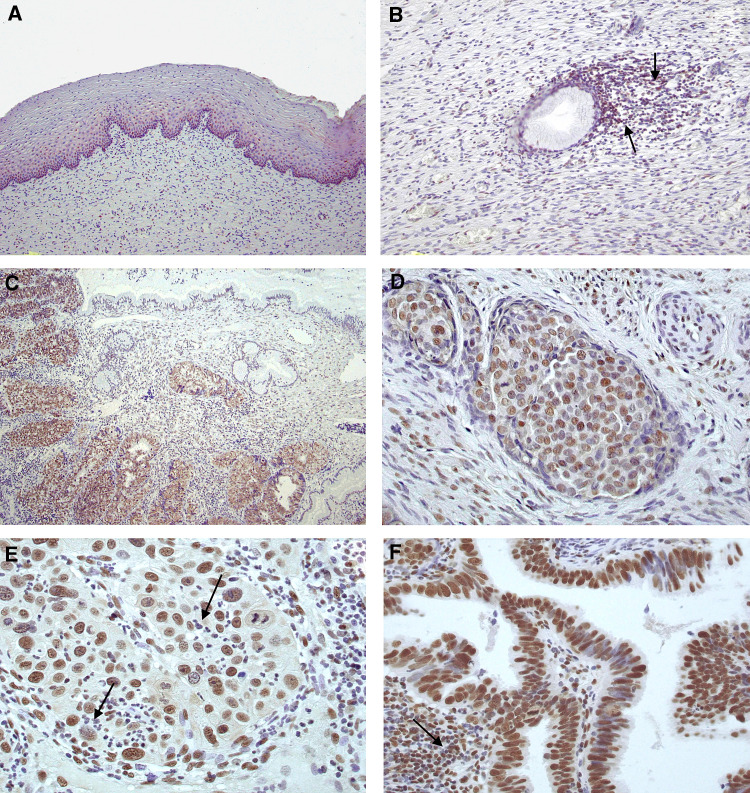
Immunohistochemical analysis of DNA-PKcs, Ku86 and Ku70 in samples from patients with cervical carcinoma. (**A**) Non-neoplastic squamous epithelium negative for DNA-PKcs protein. (**B**) Ordinary cervix gland negative for DNA-PKcs, with positively stained lymphocytes in the adjacent tissue, indicated with arrows. (**C**) Positive DNA-PKcs protein expression in partly poorly differentiated adenocarcinoma. The surrounding non-neoplastic tissue demonstrates lack of immunoreactivity concerning DNA-PKcs. (**D**) Positive expression of DNA-PKcs in squamous cell carcinoma. (**E**) Poorly differentiated squamous cell carcinoma as demonstrated by a Ku86 staining present in the tumour. Negatively stained granulocytes are indicated with arrows. (**F**) Nuclear staining of Ku70 protein in well-differentiated adenocarcinoma. Majority of cells are positively stained with high intensity. Inflammatory cell reaction with small lymphocytes expressing Ku70 (arrow).

Immunostaining was performed on formalin-fixed biopsies against DNA-PKcs (Figure 1A–D), Ku86 (Figure 1E) and Ku70 (Figure 1F). Non-neoplastic squamous epithelium and tumour-free cervical glands were negative for all of the DNA-PK subunits. In tumour-transformed tissue, nuclear staining of DNA-PKcs could be detected in all of the 109 tumours analysed. The percentage of DNA-PKcs positive cells within a tumour sample varied between 20 and 100%, with a median of 66%. DNA-PKcs was expressed with varying intensity between tumour samples. The majority of the samples were intermediately or strongly positive for DNA-PKcs. In 10 samples the intensity was judged as low. No significant difference in expression of DNA-PK between small/intermediate and large tumours were found. Staining for Ku86 was found in 107 cases and the percentage of positive cells varied between 24 and 100%, with a median of 74%. Expression of Ku70 was detected in all but one of the tumours analysed. The percentage immunopositive cells ranged from 15 to 99%, with a median of 78%. For both Ku86 and Ku70 the immunostaining were localised to the nucleus and the intensity of staining were judged as strong. When comparing small/intermediate and large tumours no significant difference in expression of neither Ku70 nor Ku86 could be found. DNA-PKcs positivity was found to correlate with Ku86 positivity (*r*_s_=0.49, *P*<0.001) as well as with Ku70 positivity (*r*_s_=0.53, *P*<0.001). In addition, a correlation between the Ku subunits (*r*_s_=0.50, *P*<0.001) was found.

Mdm-2, p53 and p21 were also assessed for on formalin-fixed primary tumour tissue. Immunostaining of Mdm-2 varied considerably between the tumours. Nine of the samples showed no staining for Mdm-2 protein. The percentage of Mdm-2 positive cells varied between 10 and 100% with a median value of 68%. In all, 59 cases displayed nuclear staining, while in 41 of the tumours Mdm-2 could only be detected in the cytoplasm. Intensity of staining was intermediate or low in 40 and 36 cases, respectively, whereas 24 cases were strongly positive for Mdm-2 protein. Immunopositivity for p53 was found in 38 of the tumours analysed and the percentage of stained cells ranged from 5 to 33%. The median value was 0 due to the high percentage of p53 negativity. Staining was nuclear, of intermediate or high intensity. Immunopositivity for p21 was found in 73 cases and the percentage stained cells ranged from 5 to 66% with a median value of 15 %. A positive correlation was found between protein expression of p53 and p21 (*r*_s_=0.27, *P*=0.005) as well as between p53 and Mdm-2 (*r*_s_=0.27, *P*=0.004) When the frequency of p53 positivity was compared between small/intermediate tumours and large tumours, the amount of cells staining positive for p53 was found to be significantly higher in large tumours (*P*=0.018). In addition, a positive correlation between tumour size and immunopositivity for p21 was also found (*P*=0.002). No correlation could, however, be found between tumour size and high and low expression of Mdm-2. p53-positive tumours showed a significantly higher expression of DNA-PKcs than p53-negative tumours (*P*=0.009). No significant difference in protein expression was detected between the different histogenetic types of cervical carcinoma. In addition, differences in protein expression between grades of differentiation could not be found. No correlation was found between long-term survival and expression of any of the analysed proteins (Tables 2 and 3).

### RT-induced remission and expression of DNA damage-related proteins

**Figure 2:**
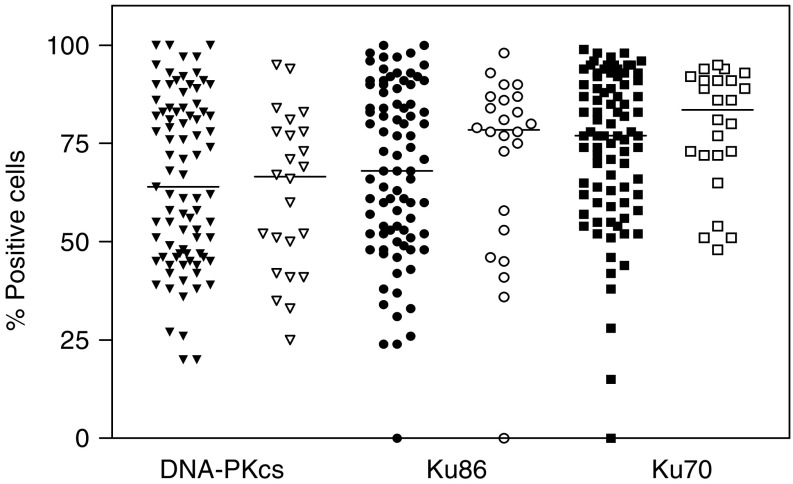
Expression of DNA-PKcs (triangles), Ku86 (circles) and Ku70 (squares) in primary biopsies from patients with cervical carcinoma and comparison between pCR (filled symbols) and non-pCR cases (unfilled symbols). Median values are indicated. NS (Mann–Whitney Test).

**Figure 3:**
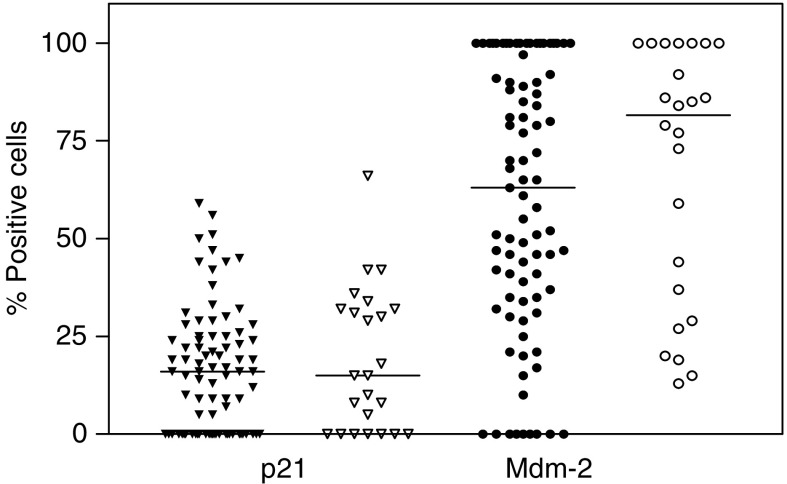
Expression of p21 (triangles) and Mdm-2 (circles) in primary biopsies from patients with cervical carcinoma and comparison between pCR (filled symbols) and non-pCR cases (unfilled symbols). Median values are indicated. NS (Mann–Whitney Test).

With an attempt to identify predictive markers for radiosensitivity, we assessed for correlations between protein expression in primary tumour and treatment outcome assessed in the surgical specimen. Comparison between pCR and non-pCR with respect to expression of DNA-PKcs, Ku86 and Ku70 were based on samples with low percentage stained cells (<median value) or high percentage stained cells (>median value). We could not detect any significant difference in protein expression of DNA-PKcs in primary tumour between pCR and non-pCR cases. In addition there were no significant difference in protein expression of the subunits Ku70 and Ku86 between pCR and non-pCR cases (Figure 2). Comparison between samples with high and low expression revealed that neither Mdm-2, nor p21 can be used as markers of early pathological response after RT in early stages of cervical carcinoma (Figure 3). There was no significant difference between nuclear and cytoplasmic Mdm-2 staining with respect to early pathological response. We found that positive p53 tumours were significantly more common among non-pCR cases compared with patients showing pCR (*P*=0.031) (Table 4). Differences in intensity of staining could not be detected for any of the analysed proteins when comparing pCR and non-pCR samples.

## DISCUSSION

The aim of our study was to see if there is a relation between DNA-PKcs and response to RT in homogenously treated cervical carcinoma of stage IB-IIA as assessed by IHC in the primary tumour. We were, however, not able to demonstrate such a correlation. In addition, related proteins as Ku70 and Ku86 also did not correlate with RT response. Among down streams effectors analysed like Mdm-2, p53 and p21 only the frequency of IHC determined p53-positive cells showed a statistically significant correlation with response to RT measured as pathological complete tumour response.

When assessing DNA-PK protein expression in primary tumour biopsies from patients with cervical carcinoma, we found non-neoplastic tissue including non-neoplastic squamous epithelium and connective tissue to express no or very low DNA-PKcs, Ku86 and Ku70. In tumour-transformed tissue in both squamous cell carcinoma and adenocarcinoma, on the other hand, positive immunostaining was found for all three proteins but to a variable extent. This is in agreement with other studies, in which DNA-PKcs, Ku86 and Ku70 were found to be upregulated in tumour tissue from bladder and colorectal carcinoma, as compared with the corresponding non-tumour tissue (Stronati *et al*, 2001; Hosoi *et al*, 2004). It has also been shown that DNA damage response proteins, such as ATM, Chk2, p53 and *γ*-H2AX are constitutively activated and expressed at higher levels during early tumorigenesis as compared to normal tissue (Bartkova *et al*, 2005).

The absence of a correlation between IHC measured expression of DNA-PK and tumour response is at some variance with other reports indicating a positive role of DNA-PK as a predictive marker for radiosensitivity in cervical carcinoma. In a study by Wilson *et al* (2000) including patients with cervical carcinoma stage I–III, all tumours with a low frequency of Ku70 or Ku80 immunopositive cells (<60%) were radiosensitive in a clonogenic assay. There was, however, no overall statistically significant correlation between *in vitro* radiosensitivity (SF_2_-values) of explanted tumour cells and expression of Ku70 or Ku80 in primary biopsies. A significant higher survival rate was found for patients whose tumours had a low level of Ku70 expression but no relation was found with low Ku80 expression. In contrast to Wilson's study, our study only included patients with early stages (IB–IIA) of cervical carcinoma. We did not find a correlation between DNA-PKcs and their subunits with response to RT. In addition, we did not find a correlation between expression in primary tumour biopsies and long-term survival for any of the studied proteins.

Differences in patient populations concerning tumour stages may be of importance for diverging results since it is not clear if or how the molecular pattern in tumours changes with progressive disease. In addition, the definition of response to RT differs between studies. As surgery was included in the treatment schedule in our study we based the definition of treatment response on the pathological evaluation of the whole cervix. Defining response to RT in the individual patient by *in vitro* methods such as soft agar clonogenic assay could be questioned since methodological factors may have a considerable effect on the SF_2_-values of explanted tumour cells which has to be accounted for when the results are interpreted. Using survival as an end point when evaluating molecular factors of importance for clinical response to RT, also has drawbacks. Survival is a variable that strongly is affected by tumour stage, age, performance status, intercurrent diseases and all treatment given to the patient. The comparison of tumour response to expression of DNA-PK seems less biased.

Harima *et al* (2003) reports significantly better clinical response to RT among Ku80-negative tumours, assessed by IHC on primary biopsies, as compared to Ku80-positive tumours. Furthermore, patients with Ku80-negative tumours were also found to have better survival than those with Ku80-positive tumours. The study included patients with microinvasive disease (FIGO stage IA) as well as patients with advanced disease and distant metastases (FIGO stage IVB) and clinical response and survival was used as a measure of radiosensitivity. Clinical response used as an end point for analysing the importance of molecular markers for treatment response can be problematic. This is especially apparent when the studied patients are heterogeneous with respect to tumour stage. Clinical response is primarily based on physical and radiological examinations, and time of evaluation, experience of the clinician as well as the type and quality of performed radiology are factors that will influence the judgement. Harima's study differed from ours with respect to mean age of the patient population (65 *vs* 44 in our study) and also by that a major part of patients presented more advanced stages (stage III–IV). A radioresistant phenotype of a tumour may originate from a small population of cells possessing the prerequisite for survival after irradiation, indicating that heterogeneity of level of protein expression on the cellular level may be of importance when searching for markers that can predict tumour response. In the Harima study the IHC scoring system differs from ours with the inclusion of staining density, a parameter which we have left out. In our study, no differences in intensity of staining could be detected for any of the analysed proteins when comparing pCR and non-pCR samples.

When assessing molecular factors important for response of tumours to RT it is important to consider the impact of FIGO-stage and tumour size (Eifel *et al*, 1994; Perez *et al*, 1998). We previously showed that small tumours more often reach complete remission following brachytherapy as compared with large tumours (Beskow *et al*, 2002). This may reflect difficulties in dose planning and targeting large tumour volumes. However, one may hypothesise that large tumours may have a different molecular pattern that could be of importance for the response to radiation. There are clinical studies on DNA-PK and outcome of RT on other tumour sites with diverging results. In head and neck cancer there are reports of no correlation between expression of DNA-PKcs and the subunits Ku70/Ku86 and *in vitro* radiosensitivity of explanted tumours (Björk-Eriksson *et al*, 1999), while in another study tumours with high levels of Ku86 displayed better locoregional control after RT as compared with tumours expressing low levels of Ku86 (Friesland *et al*, 2003). In a study of patients with rectal carcinoma treated with preoperative RT, low levels of Ku70 immunostaining was related to radiosensitivity (Komuro *et al*, 2003). It is known that deficiency in any of the DNA-PK subunits leads to impaired DNA DSB repair and increased sensitivity to ionising radiation (Taccioli *et al*, 1994; Jeggo *et al*, 1995; Kirchgessner *et al*, 1995). Also, activity and DNA-PK expression has been shown to correlate with radiosensitivity in both lung cancer cell lines (Sirzén *et al*, 1999) and oesophageal cancer cell lines (Zhao *et al*, 2000). However, the significance of DNA-PK in biopsy specimen as a predictive indicator of radiosensitivity is clearly not as obvious. A possible explanation for this diversity could be the heterogeneity of a tumour as compared to cell lines. Other factors of importance may be the surrounding tissue of a tumour, which may influence the tumour response to treatment, or the degree of hypoxia. One also has to consider the possibility that non-pCR cases represent a group of cases that vary in sensitivity to RT since we were not able to assess the exact decrease of tumour mass but just divide the material in pCR and non-pCR. The total overlap between the groups as regards IHC positivity, however, argues against such an explanation for the lack of difference between the pCR and non-pCR groups.

Prognostic significance for p53 immunostaining has previously been evaluated for RT in patients with cervical carcinoma (Ebara *et al*, 1996; Mukherjee *et al*, 2001). In primary biopsies from patients with advanced cervical carcinoma, p53 protein expression was not found to correlate with treatment outcome (Ebara *et al*, 1996). We did, however, find that primary tumour biopsies staining positive for p53 will confer non-pCR, which is in agreement with the findings by (Mukherjee *et al*, 2001). In cervical carcinoma, there are two different mechanisms that may explain the loss of p53 function: a somatic gene mutation, which leads to an inactive form, and the enhanced protein degradation promoted by the E6 oncoprotein of the human papilloma virus types (HPV) 16 and 18 (Scheffner *et al*, 1990). In contrast to many other human tumour forms, p53 mutations are only rarely detected in cervical carcinoma (Benjamin *et al*, 1996). In our study, we do not know if the E6-mediated pathway is responsible for the detected correlation between p53 positivity and lack of tumour response to RT. There is, however, evidence showing that expression of p53 does not differ between HPV-positive and -negative cervical cancers (Troncone *et al*, 1998). On the other hand, it is difficult to conclude that mutant p53 is the underlying cause of radioresistance in cervical carcinoma. The p53 antibody used in this study reacts with an epitope in the N-terminus of both wild and mutant types of p53 protein localised in the cell nucleus. Also, it has been shown that p53 mutation and immunoreactivity, cannot be correlated in cervical carcinoma, which is in contrast to mammary and endometrial cancer (Schneider *et al*, 1994).

In conclusion, results of the present study suggest a role for DNA-PK in tumour transformation since elevated levels of DNA-PKcs, Ku86 and Ku70 were found in tumour tissue as compared with non-neoplastic tissue. Our results, however, imply that immunohistochemical assessment of the DNA-PK complex cannot be used as a predictive marker for clinical response to brachytherapy of early stages of cervical carcinoma. However, expression of p53 protein is more frequent among cases with incomplete pathological remission after brachytherapy of cervical carcinoma.

## Figures and Tables

**Table 1:** Tumour characteristics in relation to tumour remission

**Parameter**	**Pathological complete remission (pCR)**	**Incomplete pathological remission (non-pCR)**	**Total no. of patients**	***P*-value**
No. of patients	85 (78%)	24 (22%)	109	
*FIGO stage*				
IB	69 (81%)	16 (19%)	85	*P*=0.130^a^
IIA	16 (67%)	8 (33%)	24	
				
*Tumour size*				
< 2 cm	24 (96%)	1 (4%)	25	
2 -<4 cm	32 (86%)	5 (14%)	37	*P*<0.001^b^
⩾4 cm	29 (62%)	18 (38%)	47	
				
*Histologic type*				
Squamous	61 (79%)	16 (21%)	77	
Adeno	19 (73%)	7 (27%)	26	*P*=0.628^a^
Adenosquamous	5 (83%)	1 (17%)	6	
				
*Grade of differentiation*				
Well	9 (69%)	4 (31%)	13	
Moderate	44 (83%)	9 (17%)	53	*P*=0.391^a^
Poor	27 (73%)	10 (27%)	37	
Unspecified	5 (83%)	1 (17%)	6	

a *χ*^2^ test.

b *χ*^2^ test for trend.

**Table 2:** Protein expression in primary tumour with respect to survival

**Factor**	**RH**	**CI**	***P*-value**
DNA-PKcs	0.995	0.975–1.015	0.606
Ku70	0.994	0.973–1.015	0.564
Ku86	0.999	0.981–1.018	0.948
Mdm2	1.004	0.990–1.017	0.599
p21	1.012	0.987–1.038	0.362

RH=relative hazard; CI=95% confidence interval.

**Table 3:** Survival and expression of p53 in primary tumour

	**No. of patients**	**Deaths**	**%**	***P*-value**
*p53*				
0	71	11	15.5	0.157
1	38	10	26.3	

**Table 4:** Staining of p53 in primary biopsies in relation to radiation response

	**p53 negative**	**p53 positive**	**No. of patients**	***P* a**
pCR^b^	60 (71%)	25 (29%)	85 (100%)	
non-pCR^c^	11 (46%)	13 (55%)	24 (100%)	0.031
Total	71	38	109	

a *P*=statistical significance using *χ*^2^ test.

b pCR=pathological complete remission.

c non-pCR=incomplete pathological remission.
